# Delivery of a chromosomally normal child from an oocyte with reciprocal aneuploid polar bodies

**DOI:** 10.1007/s10815-012-9746-6

**Published:** 2012-03-30

**Authors:** Richard T. Scott, Nathan R. Treff, John Stevens, Eric J. Forman, Kathleen H. Hong, Mandy G. Katz-Jaffe, William B. Schoolcraft

**Affiliations:** 1 Reproductive Medicine Associates of New Jersey, 111 Madison Ave, Morristown, NJ 07960 USA; 2 UMDNJ-Robert Wood Johnson Medical School, 125 Paterson Street, New Brunswick, NJ 08901 USA; 3 Rutgers-The State University of New Jersey, 145 Bevier Road, Piscataway, NJ 08854 USA; 4 Colorado Center for Reproductive Medicine, 10290 RidgeGate Circle, Lone Tree, CO 80124 USA

**Keywords:** Polar body, SNP microarray, Meiosis, Aneuploidy, Comprehensive chromosome screening

## Abstract

**Purpose:**

To demonstrate that a euploid embryo derived from an oocyte with reciprocal aneuploid polar bodies is capable of producing a chromosomally normal child.

**Methods:**

A case report of maternal MI error compensation where single nucleotide polymorphism (SNP) microarray based comprehensive chromosome screening (CCS) was performed on the 1st and 2nd polar body, the resulting embryo, and newborn DNA.

**Results:**

CCS performed after embryo transfer identified a chromosomally normal embryo that resulted from an oocyte with reciprocal aneuploid polar bodies. The first polar body was found to be missing a single chromatid derived from chromosome 21 and the second polar body possessed an extra chromatid derived from chromosome 21. Compensation of the maternal meiotic error was verified by CCS analysis of a trophectoderm biopsy from the resulting blastocyst which was euploid for all 23 pairs of chromosomes. DNA fingerprinting and CCS of the resulting newborn confirmed a chromosomally normal child, demonstrating the developmental potential of an oocyte with reciprocal aneuploid polar bodies.

**Conclusions:**

This is the first case report demonstrating the reproductive potential of a chromosomally normal embryo derived from an oocyte which had undergone meiosis I error. Systematic investigation into the frequency of meiosis I error compensation and the negative predictive value of polar body aneuploidy screening for reproductive potential should be conducted in order to confirm clinical relevance.

## Introduction

There is growing interest in the development of new comprehensive methods of 24-chromosome aneuploidy screening (CCS) to reduce the time to pregnancy and the incidence of miscarriage in patients with infertility. Indeed, preclinical validation of array based methodologies have been encouraging [1–6] and clinical results of comprehensive methods have been promising [7, 8]. These studies have now led to the development of a number of randomized controlled trials (RCTs) to assess clinical efficacy. Most of the ongoing RCTs (www.clinicaltrials.gov ID numbers, NCT01194531, NCT01219283, and NCT01332643, and www.controlled-trials.com ID number ISRCTN37972669) involve evaluation of array comparative genomic hybridization (aCGH) or single nucleotide polymorphism (SNP) array technology of embryonic biopsies (cleavage or blastocyst). In contrast, the European Society of Human Reproduction and Embryology (ESHRE) Preimplantation Genetic Screening (PGS) Task Force has initiated a multicenter RCT to characterize the utility of polar body aCGH [9]. Each approach has a variety of theoretical limitations that are both technical and biological in nature [10].

One challenge associated with polar body chromosome screening is related to making the decision to discard oocytes with aneuploid polar bodies or based on aneuploidy in the 1st polar body alone [11, 12]. In a preclinical study, the ESHRE PGS Task Force aCGH data indicated that 6 of the 138 oocyte/polar body pairs (4 %) displayed euploidy in the resulting zygotes despite aneuploidy in the corresponding polar bodies [5]. Another study, using polar body metaphase (m)CGH, reported a similar phenomenon at a frequency of 1.7 % (2 of 113) [13]. In the latter study the errors could be explained through compensation of meiosis I premature separation of sister chromatids (PSSC) during meiosis II. Specifically, when the 1st polar body acquired only one chromatid from meiosis I and the 2nd polar body acquired the corresponding extra chromatid from meiosis II, the resulting zygote could be euploid. Likewise, an extra chromatid in the 1st polar body and a corresponding missing chromatid in the 2nd polar body could also lead to a euploid zygote. However, the ability of euploid zygotes derived from oocytes with reciprocal aneuploid polar bodies to produce healthy offspring is unknown. This case report presents the first evidence to support the potential for reproductive competence of oocytes with reciprocal aneuploid polar bodies.

## Materials and methods

This specific case came from a clinical study to determine the predictive value of DNA fingerprinting of polar bodies and embryonic cells (ClinicalTrials.gov ID# NCT01219517). This study was performed with institutional review board approval and patient consent. A couple presenting for infertility treatment based on advanced maternal age (41 years) with normal ovarian reserve and no male factor infertility was recruited and consented into this IRB approved study. The female partner underwent a down regulation antagonist ovarian stimulation protocol. A total of 16 oocytes were retrieved, 11 matured and 10 fertilized by intracytoplasmic sperm injection. All fertilized zygotes were cultured in sequential media to the blastocyst stage following 1st (Day 0) and 2nd (Day 1) polar body biopsy as previously described [14]. A trophectoderm biopsy was performed on day 5 of embryonic development as previously described [7] prior to a fresh day 5 blastocyst transfer. A twin clinical pregnancy was confirmed by ultrasound with fetal heart tones. Both female babies were delivered liveborn with no complications.

The case was identified using a validated method of CCS involving whole genome amplification and SNP microarray analysis on sequentially biopsied 1st and 2nd polar bodies, and trophectoderm cells as previously described [1, 7, 14]. Upon delivery, newborn buccal DNA was obtained to determine the chromosome constitution and to perform DNA fingerprinting to confirm genetic identity to the 1st polar body and trophectoderm of the transferred embryo as previously described [14–16]. First polar bodies from sibling oocytes and trophectoderm from supernumerary cryo-preserved (non-transferred) sibling embryos were used as controls for predicting the relationship between each of the newborn females. For 1st polar body based fingerprinting, a 40 % threshold was applied providing 100 % sensitivity and specificity for distinguishing siblings and identifying identical relationships from the 1st polar body as previously published [15]. First polar bodies with less than 40 % identity to newborn DNA are considered self relationships. For trophectoderm biopsy based fingerprinting, a 50 % threshold was applied based on a similar 100 % predictive value for distinguishing siblings and identifying identical relationships from a single blastomere or trophectoderm biopsy [16]. Trophectoderm with greater than 50 % identity to newborn DNA are considered self relationships.

## Results

**Fig. 1:**
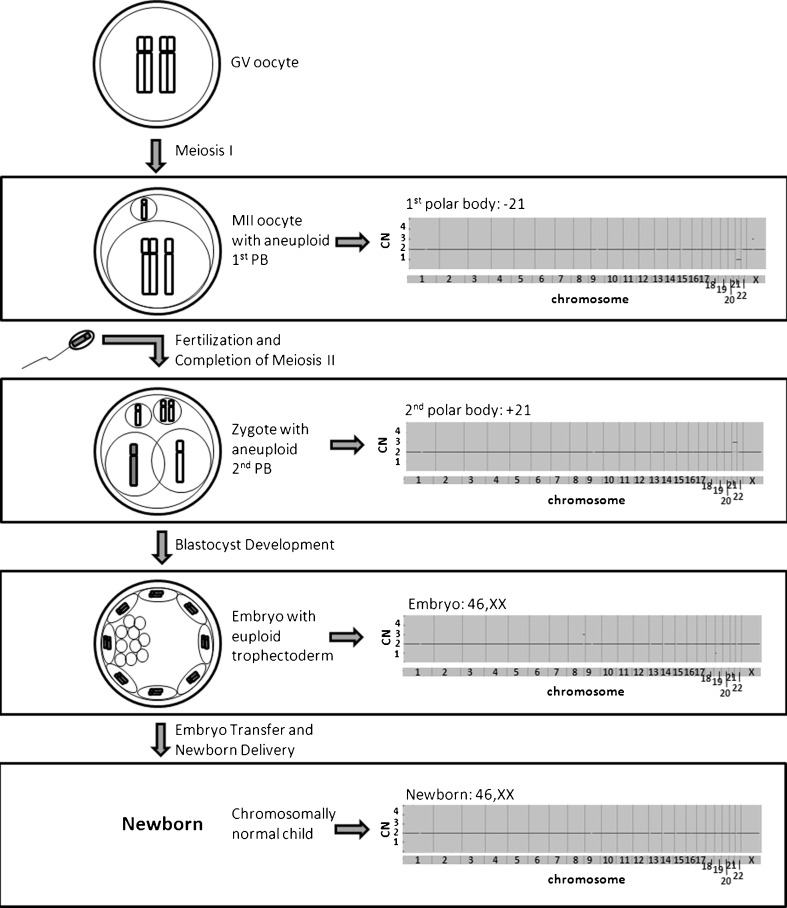
An oocyte with reciprocal aneuploid polar bodies is capable of producing a chromosomally normal child. Diagrams (*left*) indicate the segregation pattern for chromosome 21 with the corresponding SNP microarray based copy number plots (*right*) of the oocyte’s 1st and 2nd polar body, a trophectoderm biopsy from the resulting embryo, and buccal cells from the newborn derived after embryo transfer. GV = germinal vesicle, PB = polar body, CN = copy number

The 1st polar body was identified as possessing a loss of genetic material from chromosome 21 (Fig. 1). The 2nd polar body was found to possess a gain of genetic material from chromosome 21 (Fig. 1). All remaining chromosomes in both polar bodies were observed as euploid. Analysis of the resulting blastocyst stage embryo trophectoderm biopsy revealed euploidy for all 23 pairs of chromosomes (Fig. 1). The pattern of copy number in the three samples from this oocyte/embryo was consistent with PSSC of chromosome 21 during meiosis I, with subsequent compensation by meiosis II segregation of the extra chromatid to the 2nd polar body rather than the remaining oocyte. Delivery of a healthy female with a normal number of chromosomes was confirmed by CCS analysis of buccal cells from the newborn (Fig. 1).

**Fig. 2:**
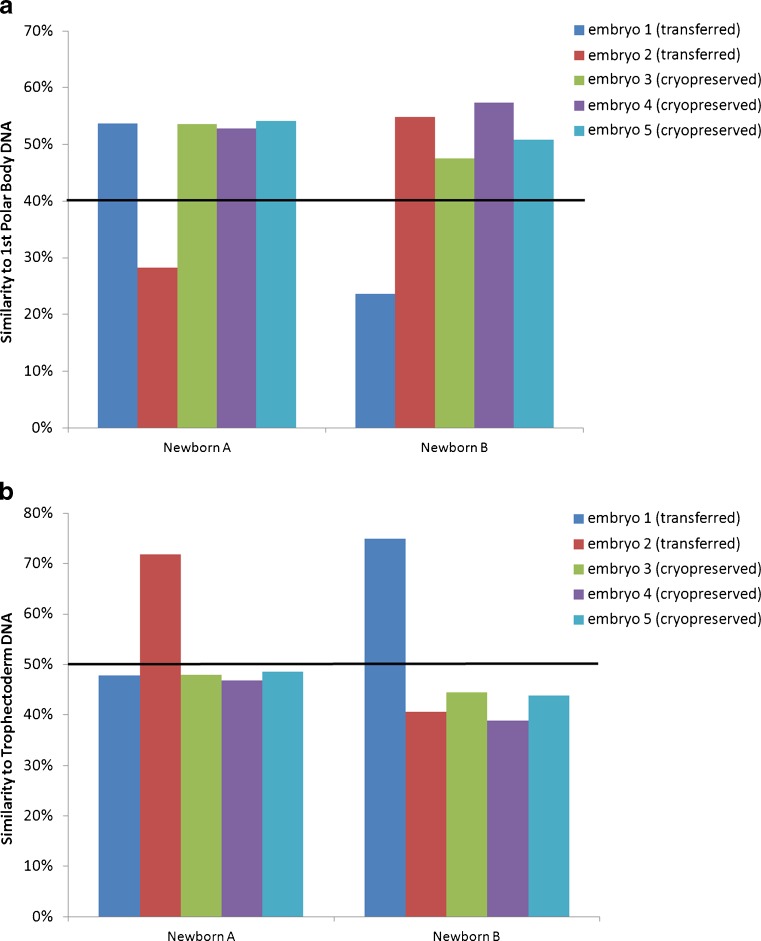
Results of genetic fingerprinting to confirm the preimplantation genetic origin of newborn DNA. **a** 1st polar body DNA based fingerprinting illustrating a match between transferred embryo #1 and newborn B, and transferred embryo #2 and newborn A. The cutoff of 40 % is indicated with a black bar and is based on previous publication [15]. Similarities below 40 % are considered a match and above a sibling. **b** Trophectoderm DNA based fingerprinting illustrating a match between transferred embryo #1 and newborn B, and transferred embryo #2 and newborn A. The cutoff of 50 % is indicated with a black bar and is based on previous publication [16]. Similarities above 50 % are considered a match and below a sibling. In each type of fingerprinting, transferred embryo #1 was the one which possessed reciprocal aneuploid polar bodies, and was the oocyte/embryo that matched newborn B. Embryo #3, 4 and 5 served as known sibling control embryos since they were not transferred

In order to confirm the genetic relationships between biopsied samples and newborn DNA, two independent methods of DNA fingerprinting were employed. First, polar body and newborn buccal DNA fingerprinting confirmed that the 1st polar body from transferred embryo #1 (with loss of a single chromosome 21 chromatid) was a genetic match with one of the two newborn DNA samples, newborn “B” (Fig. 2a). Importantly, newborn B matched only this particular 1st polar body while failing to match 3 known sibling oocyte derived 1st polar bodies (embryo #3, 4, and 5) and the other sibling oocyte, embryo 2 that was transferred. The same observations were made for the relationships between the 1st polar body from transferred embryo #2 and newborn A.

As a second measure of identity, trophectoderm and newborn buccal DNA fingerprinting confirmed that transferred embryo #1 (derived from the oocyte with reciprocal aneuploid polar bodies) was a genetic match with one of the two newborn DNA samples, newborn “B” (Fig. 2b). Newborn B matched only this particular trophectoderm biopsy while failing to match 3 known sibling embryo derived trophectoderm biopsies (sibling cryopreserved embryos #3, 4, and 5) and the other transferred embryo (embryo #2). The same observations were made for the relationships between the trophectoderm from transferred embryo #2 and newborn A. Furthermore, the relationships identified between both the 1st polar bodies and the trophectoderm biopsies with newborn DNAs were 100 % consistent with each other.

## Discussion

These results provide a definitive proof-of-principle that a euploid zygote derived from an oocyte with reciprocal aneuploid polar bodies is capable of producing a chromosomally normal child. This was demonstrated with the use of a single cell CCS methodology proven to provide 98.6 % accuracy of 24 chromosome copy number assignment and no false positives [1]. In addition, it is highly unlikely that the 1st polar body would have a false positive loss of the same chromosome found to have a false positive gain in the 2nd polar body (probability of 0.03 %). Chromosome analyses demonstrated that the trophectoderm biopsy from the blastocyst and buccal cells from the newborn were euploid. Finally, a genetic match for the 1st polar body and the newborn, and for the trophectoderm biopsy and the same newborn was confirmed using two independent DNA fingerprinting methodologies that have been shown to provide a 100 % level of certainty for polar body and embryo biopsy DNA [15, 16].

Interestingly, prior to the completion of meiosis II, this oocyte was at risk of developing into an embryo with trisomy 21, with the potential to result in delivery of a child with Down syndrome. However, as a result of the extra chromatid segregating to the 2nd polar body rather than the oocyte, the subsequent embryo acquired a euploid karyotype. This may be a random event with an equal proportion of oocytes inheriting either an abnormal or normal number of chromosomes following a meiosis I PSSC error [17]. However, this has yet to be supported with experimental evidence. Alternatively, it is intriguing to hypothesize that this process could be influenced by the microenvironment of the developing oocyte towards the favorable outcome of euploidy.

Given that this case report has demonstrated the reproductive potential of a euploid embryo derived from an oocyte with reciprocal aneuploid polar bodies, it questions discarding an oocyte based solely on the observation of reciprocal aneuploidy in the polar bodies or based on identification of aneuploidy in the first polar body alone [11, 12]. Instead, analysis of aneuploidy in the resulting embryo should be performed to ascertain the frequency of development of euploid embryos from oocytes with reciprocal aneuploid polar bodies. This is particularly true given the estimate that most MI errors are from PSSC and the results of the present case report. An initial review of SNP microarray data from over 1,000 polar body pairs has identified 43 cases (~4 %) in which the 1st and 2nd polar bodies are perfectly complementary. The prevalence of euploidy in the embryos resulting from these oocytes now represents an area of active ongoing research in our own laboratories in order to estimate the clinical relevance of this phenomenon.
